# Capacitive Behavior of Single Gallium Oxide Nanobelt

**DOI:** 10.3390/ma8085244

**Published:** 2015-08-17

**Authors:** Haitao Cai, Hang Liu, Huichao Zhu, Pai Shao, Changmin Hou

**Affiliations:** 1 School of Electronic Science and Technology, Key Laboratory for Integrated Circuits Technology of Liaoning Province, Dalian University of Technology, Dalian 116024, China; E-Mails: caihaitao2000@mail.dlut.edu.cn (H.C.); liuhang@dlut.edu.cn (H.L.); shaopai@gwu.edu (P.S.); 2 State Key Laboratory of Inorganic Synthesis and Preparative Chemistry, College of Chemistry, Jilin University, Changchun 130012, China

**Keywords:** Ga_2_O_3_, nanobelt, capacitive behavior, impedance analysis

## Abstract

In this research, monocrystalline gallium oxide (Ga_2_O_3_) nanobelts were synthesized through oxidation of metal gallium at high temperature. An electronic device, based on an individual Ga_2_O_3_ nanobelt on Pt interdigital electrodes (IDEs), was fabricated to investigate the electrical characteristics of the Ga_2_O_3_ nanobelt in a dry atmosphere at room temperature. The current-voltage (I-V) and I/V-t characteristics show the capacitive behavior of the Ga_2_O_3_ nanobelt, indicating the existence of capacitive elements in the Pt/Ga_2_O_3_/Pt structure.

## 1. Introduction

As is well known, low dimensional metal oxide nanostructures such as nanoparticle, nanorod, nanowire, nanoribbon, nanobelt and nanosheet have been considered as ideal candidates for various novel electronic and optoelectronic devices due to their ultra-large surface to volume ratio and novel size effect. Among them, because of having a large band gap of 4.9 eV and thus a unique transparency from visible into ultraviolet (UV) region with a cut-off wavelength of ~260 nm [1], monoclinic gallium oxide (β-Ga_2_O_3_) may, therefore, become an attractive material for future generations of optoelectronic devices operating at shorter wavelengths where standard transparent conducting oxides (TCOs) such as indium-tin oxide (ITO) are already non-transparent [2,3,4,5,6]. Moreover, due to its good chemical and thermal stability and widely tunable properties, Ga_2_O_3_ has wide applications in insulating barriers for spin-dependent tunneling junctions [7], electroluminescent phosphors [8,9], field emission devices [10], field effect transistors [11,12], resistance switching memories [13,14] and gas sensors [15,16,17,18,19,20,21]. Among all these reports about the novel electrical and optoelectrical properties of Ga_2_O_3_, mention of its capacitive property is very infrequent. In the present study, we synthesized monocrystalline Ga_2_O_3_ nanobelts through a chemical vapor deposition (CVD) process and fabricated a simple electronic device based on them. Interestingly, we observed a capacitive behavior from an individual Ga_2_O_3_ nanobelt on Pt interdigital electrodes and reported here for the first time. The crystalline structure of the grown Ga_2_O_3_ nanobelt was analysed by an X-ray powder diffractometer (XRD, D8 Advance, Bruker Corporation, Karlsruhe, Germany, Cu Kα radiation with λ = 1.5406 Å). The goniometer scanning rate was 0.4°·min^−1^. The morphology and structure of as-grown Ga_2_O_3_ nanobelt was investigated through a scanning electron microscope (SEM, JSM-6700F, JEOL, Tokyo, Japan) and a high-resolution transmission electron microscope (TEM, Tecnai G^2^ F20 S-TWIN, FEI Company, Hillsboro, OR, USA, working at 200 kV with a LaB_6_ filament).

## 2. Results and Discussion

**Figure 1:**
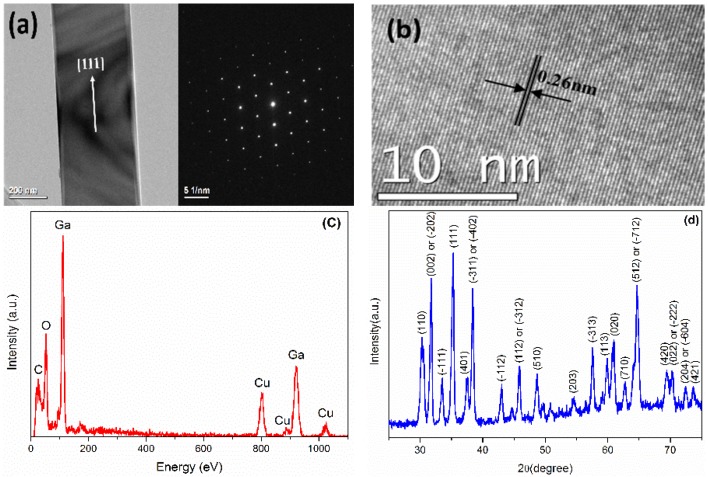
(**a**,**b**) transmission electron microscope (TEM) images, (**c**) energy dispersive X-ray spectrometry (EDS) spectrum and (**d**) X-ray powder diffractometer (XRD) spectrum of Ga_2_O_3_ nanobelts.

The structure of the Ga_2_O_3_ nanobelts was investigated using a Tecnai G^2^ F20 transmission electron microscope (TEM) (FEI Company), and the results of an individual nanobelt are shown in Figure 1a,b. The low magnification TEM image shows the very thin thickness of an individual Ga_2_O_3_ nanobelt, and the high magnification TEM image and selected area electron diffraction (SAED) image confirm it has a monocrystalline structure. The distance between two consecutive planes is ~0.26 nm, confirming the growth direction of Ga_2_O_3_ nanobelt as in [111] [22]. Figure 1c shows the energy dispersive X-ray spectrometry (EDS) spectrum and Figure 1d shows the XRD spectrum of the Ga_2_O_3_ nanobelts, respectively. The diffraction peaks positions are in good agreement with those for monoclinic β-Ga_2_O_3_ powder recorded in the powder diffraction file database (powder diffraction file (PDF #760573), inorganic crystal structure database (ICSD #034243)). The relative intensities of these peaks are not perfectly consistent with those of the bulk Ga_2_O_3_, which may frequently happen for nanostructures, and is understandable because of the size-effect and distribution disorder of the nanobelts.

**Figure 2:**
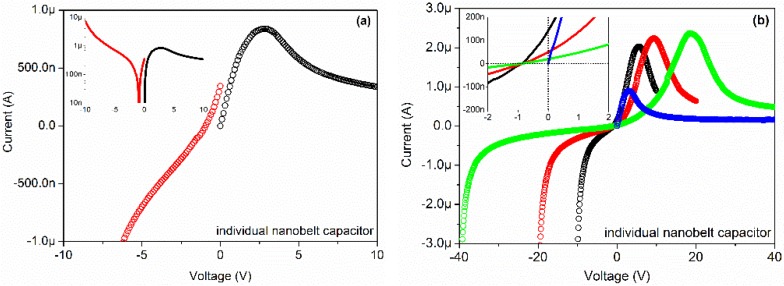
Current-voltage (I-V) characteristics of the individual Ga_2_O_3_ nanobelt on Pt interdigital electrodes (IDEs).

The current-voltage (I-V) characteristics of the device were studied by direct current (DC) voltage sweep measurements and the results are illustrated in Figure 2a. The inset map shows the same data in semi-log plot. Two voltage sweep measurements were applied on the device successively. The first voltage sweep measurement was from −10 to 0 V with steps of 0.1 V (red circle), and the second voltage sweep measurement was from 0 to +10 V with steps of 0.1 V (black circle). As the first applied voltage was swept from −10 to around −1 V, an evident decrease of the current from −10 μA to 0 A was observed. With further decrease of the applied voltage from around −1 to 0 V, an interesting positive current from 0 A to 400 nA was observed, which is a fingerprint-like proof of the discharging character of capacitors. As the second applied voltage was swept from 0 to +2.5 V, an increase in the current from 0 A to 800 nA was observed. With further increase of the applied voltage from +2.5 to +10 V, a strange gradual decrease of the current from 800 to 380 nA was observed, which is a fingerprint-like proof of the charging character of capacitors. The first voltage sweep process was the equivalent to charging the device with a linearly varying voltage from high value to low value in a short time (100 s). In this process, the discharging phenomenon happened as the charging voltage became small enough, which is the reason for reverse current observed in the device. The second voltage sweep process was the equivalent to charging the device with a linearly varying voltage from low value to high value in a short time (100 s). In this process, the charging current first rapidly increased from zero to a high value and then slowly decreased to a saturate value as the capacitors were nearly full.

Another four voltage sweeps with steps of 0.1 V were applied on the device successively, as Figure 2b shows. The inset map shows the same data in detail. The sweeps ranged from −10 to 0 to +10 V (black), from −20 to 0 to +20 V (red), from −40 to 0 to +40 V (blue), from 0 to +40 V (green), respectively. As shown in the figure, the voltage sweeps from negative to zero to positive lead to a similar current variation. Take the blue curve (−40 → 0 → +40 V) for example, as the applied voltage was swept from −40 to −1 V, an evident decrease of the current was observed. With further decrease of the applied voltage from −1 to 0 V, a positive current value was observed. As the applied voltage increased from 0 to +20 V, an evident increase of the current was observed. With further increases in the applied voltage from +20 to +40 V, a decrease of the current was observed. The reason for early emergence of the positive current is the same as what happened in Figure 2a. These measurements indicate the existence of capacitors in the device. An important question is, where do the capacitors originate?

**Figure 3:**
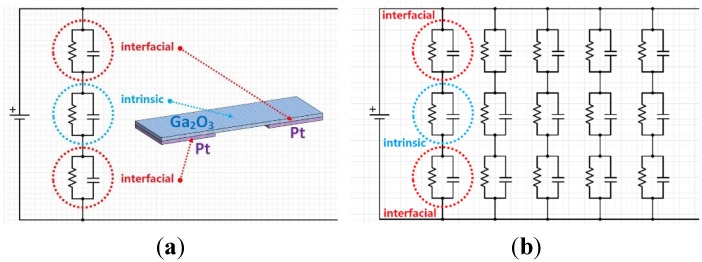
(**a**) Origin of capacitors of the individual Ga_2_O_3_ nanobelt on Pt IDEs and (**b**) the circuit model of the individual Ga_2_O_3_ nanobelt on Pt IDEs.

The possible origin of the capacitors in the individual Ga_2_O_3_ nanobelt device is shown in Figure 3. In Figure 3a, the schematic diagram of a single stage Ga_2_O_3_ nanobelt on Pt electrodes indicates that three parts may have a relationship with the impedance of the device. Namely the double interfacial parts (red circle) between Ga_2_O_3_ nanobelt and Pt, and the middle intrinsic part (blue circle) of Ga_2_O_3_ nanobelt between double Pt electrodes. Since these three parts of the single stage Ga_2_O_3_ nanobelt on Pt electrodes are in series, then their impedances should also be in series. Every part should contain a resistor and a capacitor in parallel, which originate from the Schottky barriers between Ga_2_O_3_ nanobelt and Pt, and intrinsic Ga_2_O_3_ nanobelt. Figure 3b shows the impedances of the individual Ga_2_O_3_ nanobelt on Pt interdigital electrodes. All stages of Ga_2_O_3_ nanobelt are in parallel, as well as their impedances. It should be noted that all resistors and capacitors in the figures may have different values, because various contact situation between Ga_2_O_3_ nanobelt and Pt may exist for the tiny difference in the surface of the irregular Pt interdigital electrodes.

**Figure 4:**
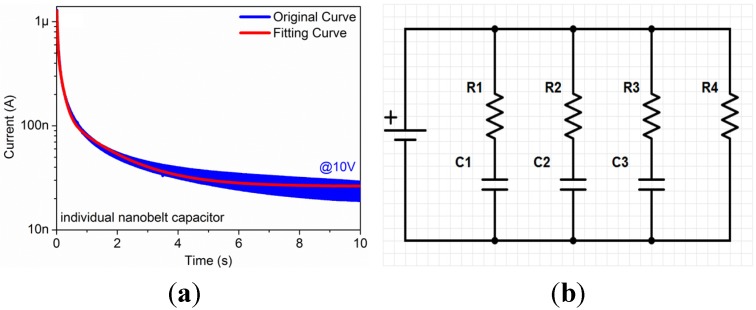
(**a**) I/V-t sweep measurement of the individual Ga_2_O_3_ nanobelt on Pt IDEs, and (**b**) simplified impedance model of the individual Ga_2_O_3_ nanobelt on Pt IDEs.

The I/V-t sweep measurement of an individual Ga_2_O_3_ nanobelt is shown in Figure 4a. The voltage was maintained constant at 10 V, and the sweep interval was maintained constant at 10 ms. Obviously, the current significantly decreased from 1.3 μA to 25 nA in 10 s, which indicates the charging characteristics of the device. Figure 4b shows a simplified impedance model of individual Ga_2_O_3_ nanobelt device according to the charging characteristics shown in Figure 4a. The red fitting curve, using a simple fitting model as Equations (1)–(5) describe as below, appears to overlap with the original blue curve perfectly. According to this fitting model, there are four resistors and three capacitors, as Equations (6)–(9) describe, located on this device:

*I*(*t*) = *I*_1_(*t*) + *I*_2_(t) + *I*_3_(t) + *I*_4_(t) (1)

*I*_1_(*t*) = (*U*/*R*_1_) × exp(−*t*/(R_1_*C*_1_)) (2)

*I*_2_(*t*) = (*U*/*R*_2_) × exp(−*t*/(*R*_2_*C*_2_)) (3)

*I*_3_(*t*) = (*U*/*R*_3_) × exp(−*t*/(*R*_3_*C*_3_)) (4)

*I*_4_(*t*) = *U*/*R*_4_, *U* = 10 V (5)

*R*_1_ = 22.3 MΩ, *C*_1_ = 7.6 nF (6)

*R*_2_ = 98.2 MΩ, *C*_2_ = 15.7 nF (7)

*R*_3_ = 8.1 MΩ, *C*_3_ = 2.9 nF (8)

*R*_4_ = 381.9 MΩ (9)

where *I* represents current, *U* represents voltage, *R* represents resistor, *C* represents capacitor, *t* represents time. Because of the complexity of impedance of this structure, it should be noted that all resistors and capacitors in this fitting model are not invariable. Once the constant voltage in I/V-t sweep measurement changed, the resistors and capacitors in the fitting model would also change, probably even their series-parallel connection situation. We consider the *R*_1_, *R*_3_, *C*_1_ and *C*_3_ are related to the interface between Pt electrodes and Ga_2_O_3_ nanobelt for the relatively small values, *R*_2_, *R*_4_ and *C*_2_ are related to intrinsic Ga_2_O_3_ nanobelt for the relatively large values.

## 3. Experimental Section

Monocrystalline Ga_2_O_3_ nanobelts were synthesized in a hot wall CVD system, using metallic gallium and oxygen as source materials. The CVD system consists of a horizontal tube furnace (600 mm in length) with a central heating zone (100 mm in length), a quartz tube connected to a gas supply and a pumping unit. Both ends of the quartz tube are sealed by rubber O-rings. The growth was controlled by a vapor-solid (VS) mechanism, in which the structural defects play an important role both during the nucleation and the preferable axial growth of nanobelts [23]. Alumina substrate was subsequently cleaned by acetone and ethanol and blown dry with synthetic air. With a high purity (99.999%) gallium grain on the surface, the alumina substrate was put in the centre of a quartz tube (600 mm in length) and transported into the tube furnace. After evacuating, the tube was filled with argon and the furnace was slowly heated up from room temperature to the growth temperature (1000 °C), during which the argon flow was maintained at 100 sccm and the pressure inside the tube was maintained at standard atmospheric pressure. When the furnace reached the target temperature, the argon flow was adjusted from 100 to 99.8 sccm and the oxygen flow was adjusted from 0 to 0.2 sccm. White wool-like products were found on the alumina substrate around the gallium metal after cooling down the system to room temperature naturally. We believe that the synthesis of Ga_2_O_3_ nanobelts resulted from the high combining ability of gallium with oxygen under high temperature.

**Figure 5:**
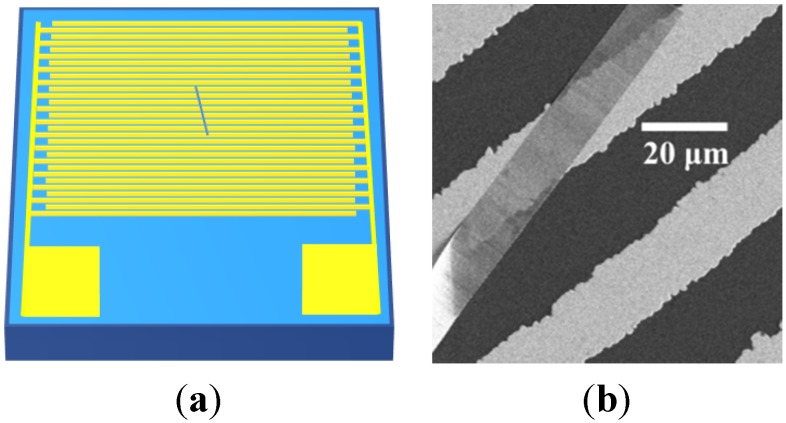
Diagram (**a**) and scanning electron microscope (SEM) image (**b**) of an individual Ga_2_O_3_ nanobelt on Pt IDEs.

A simple electronic device based on an individual Ga_2_O_3_ nanobelt for the electrical investigation was fabricated. The as-grown Ga_2_O_3_ nanobelts were initially dispersed in ethanol with the assistance of ultrasonic, and then an individual nanobelt was carefully transferred through a self-made micro-manipulating system onto a SiO_2_/Si substrate (SiO_2_ thickness: 300 nm) provided with Pt interdigital electrodes (trace 25 μm, gap 25 μm) on the surface of insulating SiO_2_ side. The diagram of individual Ga_2_O_3_ nanobelt device is shown in Figure 5. The Ga_2_O_3_ nanobelt has a large length of hundreds of microns and also a large width of ~20 microns, yet its thickness is still in nanoscale. The SEM image shows that the very thin thickness of nanobelt, for its transparency to the scanning electrons. In order to remove the residual ethanol and to ensure a good adherence of the Ga_2_O_3_ nanobelts to the substrate, a following drying process was performed. The device was placed in a drying vessel at room temperature for 12 h, followed by heating in a tube furnace at 200 °C for 2 h. The electrical measurement was performed by the B1500A semiconductor device analyzer (Agilent, Santa Clara, CA, USA) at room temperature in a synthetic dry air atmosphere containing 80% nitrogen and 20% oxygen in order to avoid any complicated influence from moisture.

## 4. Conclusions

In summary, monocrystalline gallium oxide (Ga_2_O_3_) nanobelts were synthesized through a chemical vapor deposition process. An electronic device based on individual Ga_2_O_3_ nanobelt on Pt interdigital electrodes (IDEs) was fabricated and investigated. The capacitive behavior of Ga_2_O_3_ nanobelt indicates the existence of capacitor element inside the device. The origin of these capacitors was believed to exist at the interfaces between Ga_2_O_3_ nanobelt and Pt, and also in the intrinsic Ga_2_O_3_ nanobelt. According to the impedance model, there are four resistors and three capacitors located in this device.
